# Digital Filtering of Three-Dimensional Lower Extremity Kinematics: an Assessment

**DOI:** 10.2478/hukin-2013-0065

**Published:** 2013-12-31

**Authors:** Jonathan Sinclair, Paul John Taylor, Sarah Jane Hobbs

**Affiliations:** 1 Division of Sport Exercise and Nutritional Sciences, University of Central Lancashire.; 2 School of Psychology, University of Central Lancashire.

**Keywords:** Low pass filter, kinematics, gait analysis

## Abstract

Errors in kinematic data are referred to as noise and are an undesirable portion of any waveform. Noise is typically removed using a low-pass filter which removes the high frequency components of the signal. The selection of an optimal frequency cut-off is very important when processing kinematic information and a number of techniques exists for the determination of an optimal frequency cut-off. Despite the importance of cut-off frequency to the efficacy of kinematic analyses there is currently a paucity of research examining the influence of different cut-off frequencies on the resultant 3-D kinematic waveforms and discrete parameters. Twenty participants ran at 4.0 m•s−1 as lower extremity kinematics in the sagittal, coronal and transverse planes were measured using an eight camera motion analysis system. The data were filtered at a range of cut-off frequencies and the discrete kinematic parameters were examined using repeated measures ANOVA’s. The similarity between the raw and filtered waveforms were examined using intra-class correlations. The results show that the cut-off frequency has a significant influence on the discrete kinematic measure across displacement and derivative information in all three planes of rotation. Furthermore, it was also revealed that as the cut-off frequency decreased the attenuation of the kinematic waveforms became more pronounced, particularly in the coronal and transverse planes at the second derivative. In conclusion, this investigation provides new information regarding the influence of digital filtering on lower extremity kinematics and re-emphasizes the importance of selecting the correct cut-off frequency.

## Introduction

Errors associated with the measurement of kinematic data can result from soft tissue artefact, improper digitization of retro-reflective markers and electrical interference (Winter et al., 1974). These errors are typically referred to as noise and are an undesirable portion of any kinematic waveform. Noise is traditionally lower in amplitude and associated with a different frequency range than that of the true signal (Jackson, 1979) and can typically be removed using a low-pass filter; the objective of any filtering technique is not only to attenuate noise but also to leave the true signal unaffected (Winter, 1990).

Several researchers (Enoka, 2008; Griffiths, 2006; Robertson et al., 2004; Winter, 2005) introduced the concept of frequency content in retroreflective marker information. Filters are constructed so that different elements of the frequency spectrum are attenuated. When using a low-pass filter the cut-off frequency is selected so that the lower frequencies remain yet the higher frequencies are attenuated. The fourth-order zero-lag is frequently utilized in biomechanical analyses (Yu et al., 1999). Butterworth filters yield a weighted average of data points across the kinematic waveform. This method of averaging time points means that the filtered data lags behind the raw data. To adjust for this time delay, the filter is passed through the data in the forward direction and then again in the opposite time order to yield data that is appropriately aligned temporally (Robertson and Dowling, 2003). In addition to attenuating any phase shift delay, the bi-directional filtering produces a sharper cut-off and is termed a fourth-order zero-lag shift (or zero-phase shift) filter (Smith, 1989). As the cut-off frequency increases, the influence of the filter on the data is reduced and the data will be similar to the raw signal, including some of the high frequency noise (Robertson and Dowling, 2003). Determining the most appropriate cut-off frequency is essential and necessitates knowledge of the signal’s characteristics.

**Figure 1:**
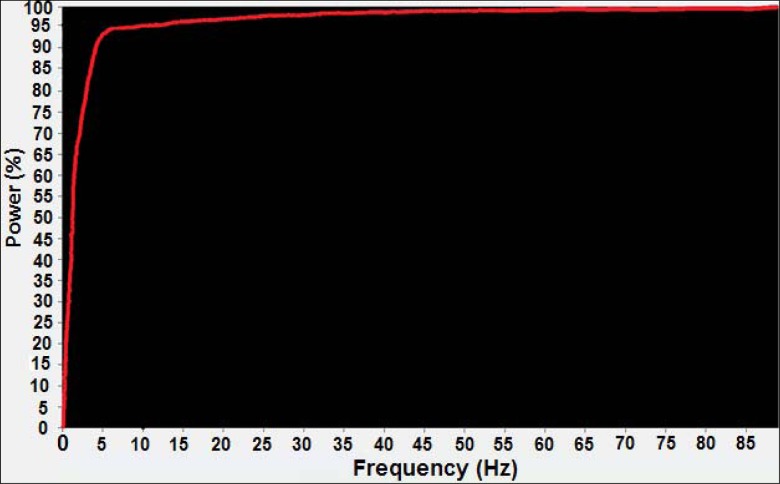
Example FFT cumulative power analysis of marker information

The selection of a frequency cut-off is very important when filtering kinematic data. A number of algorithms exists which are designed to define an objective criterion for the determination of an appropriate cut-off frequency (Jackson, 1979). The first option is to perform at spectral analysis of the marker trajectories using a fast fourier transform (FFT) to examine the cumulative content of the signal in the frequency domain (Figure 1) (Giakas and Baltzopoulos, 1997). Typically the choice of cut-off is taken as the frequency at which either 95 or 99% of the signal power is contained below (Sinclair et al., 2012, 2013a, 2013b).

**Figure 2:**
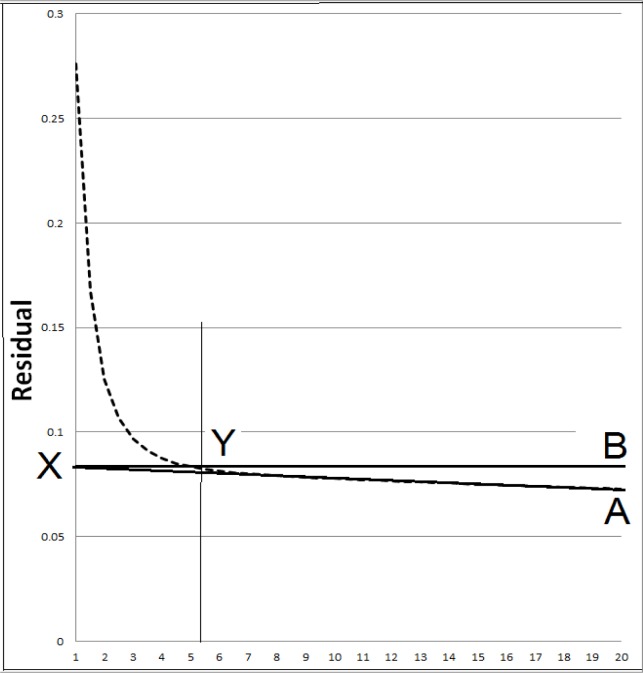
Example residual plot between filtered and unfiltered signals as a function of frequency cut-off

A second option is to perform a residual analysis (Figure 2). Residual analyses examine the differences between the raw and filtered kinematic pattern over a pre-set range of cut-off frequencies (Wells and Winter, 1980). The term residual refers to the signal content that remains when then filtered data is subtracted from the raw data (Robertson, 2005). The geometric line A represents the best estimate of the noise residual and is positioned so that it follows the linear portion of the residual plot and intercepts the Y – axis at X (0 Hz). The decision regarding the cut-off requires a compromise between the extent of signal attenuation and the amount of noise allowed to pass through. Typically a horizontal line B is projected from X to intersect the residual plot at Y and the cut-off is selected at this frequency.

Whilst it is recognised that the low-pass filter is the most commonly utilized smoothing procedure, the efficacy of the different methods for determining the optimal cut-off frequency remains unknown. The purpose of this investigation was to determine the effects of various low-pass filter cut-off’s and optimal filter techniques on kinematic waveforms and discrete parameters. This study hypothesizes that the cutoff frequency will have a significant influence on the discrete 3-D kinematic parameters.

## Material and Methods

### Participants

Twenty participants (sixteen males and four females) (age 26.33 ± 5.37 years, body height 1.76 ± 0.12 m and body mass 75.5 ± 8.6 kg) ran at 4.0m·s^−1^±5%. All were injury free at the time of data collection and provided written informed consent. Ethical approval for this project was obtained from the University of Central Lancashire School of Psychology ethics committee.

### Procedure

Participants ran at 4.0 m·s^−1^ over a force plate (Kistler, Kistler Instruments Ltd., Alton, Hampshire) embedded in the floor (Altrosports 6mm, Altro Ltd) of a 22 m biomechanics laboratory. Running velocity was quantified using Newtest 300 infrared timing gates (Newtest, Oy Koulukatu, Finland), a maximum deviation of ±5% from the set velocity was allowed. Stance time was defined as the time over which 20 N or greater of vertical force was applied to the force platform (Sinclair et al., 2011). A successful trial was defined as one within the specified velocity range, where all tracking clusters were in view of the cameras, the foot made full contact with the force plate and no evidence of gait modifications due to the experimental conditions.

Kinematic data was captured at 250 Hz via an eight camera motion analysis system (Qualisys Medical AB, Goteburg, Sweden). Calibration of the system was performed before each data collection session. Only calibrations which produced average residuals of less than 0.85 mm for each camera for a 750.5 mm wand length and points above 3000 in all cameras were accepted prior to data collection.

The marker set used for the study was based on the calibrated anatomical systems technique (CAST) (Cappozzo et al., 1995). In order to define the right; foot, shank and thigh retro-reflective markers were attached unilaterally to the calcaneus, 1st and 5th metatarsal heads, medial and lateral malleoli, medial and lateral epicondyle of the femur and greater trochanter. To define the pelvis additional retro-reflective markers were placed on the anterior (ASIS) and posterior (PSIS) superior iliac spines. Rigid tracking clusters were positioned on the shank and thigh. Each rigid cluster comprised four 19 mm diameter spherical reflective markers mounted to a thin sheath of lightweight carbon fibre with length to width ratios in accordance with Cappozzo et al. (1997). A static trial was conducted with the participant in the anatomical position in order for the positions of the anatomical markers to be referenced in relation to the tracking clusters, following which they were removed.

### Data processing

Analysis of marker displacement data from the stance phase of the running cycle were examined using both residual and FFT analyses. Marker samples beyond toe-off were ignored, thus removing any non-stance data from the fourier reconstruction. Each subsample consisted of a total of 256 values, comprising N-adjusted marker values from a given contact phase and *L* = 256-N zeros. The power (PW) of each component of the spectrum was calculated as the square of its amplitude (AM). Since the addition of zeros to the time-domain data reduces the calculated powers by a factor of *N / (N+L),* the inverse of this factor was applied to the power calculation to obtain true powers of the nonzero data.

$$\text{Thus},\;\mathrm{PW}=(\mathrm{AM})\backslash \text{N}+L)\;N^{-1}$$

The residual analysis revealed an optimal cut-off frequency of 10Hz whereas the frequency analyses revealed frequencies of 25Hz 99% signal power and 10Hz 95% signal power.

Hip, knee and ankle joint kinematics were quantified using Visual 3-D (C-Motion Inc, Germantown, USA) processed using raw data and also filtered using cut-off frequencies of 1Hz, 3Hz, 5Hz, 7Hz, 10Hz, 15Hz, 20Hz, 25Hz using a zero-lag low pass Butterworth 4^th^ order filter. Lower extremity joint angles were created using an XYZ cardan sequence of rotations (Sinclair et al., 2012). All data were normalized to 100% of the stance phase then mean processed gait trial data was reported. Displacement specific 3-D kinematic measures from the hip, knee and ankle which were extracted for statistical analysis were 1) ROM from footstrike to toe-off during stance and 2) peak angle during stance. First derivative angular velocity measures that were extracted were 1) peak stance phase angular velocity. Second derivative angular acceleration measures 1) peak stance phase angular acceleration.

### Statistical analyses

Descriptive statistics including means and standard deviations were calculated for each frequency cut-off. The statistical differences in each of the kinematic parameters as function of cut-off frequency were examined using repeated measures ANOVA’s with significance accepted at the (p≤0.05) level. Post-hoc pairwise comparisons were utilized for all significant main effects using a Bonferroni adjustment to control type I error. In addition to compare the similarity between the raw and filtered stance phase waveforms (i.e. extent of signal distortion/ attenuation) each of the mean filtered stance phase curves were contrasted against the normalized unfiltered waveform using intra-class correlations.

## Results

**Figure 3:**
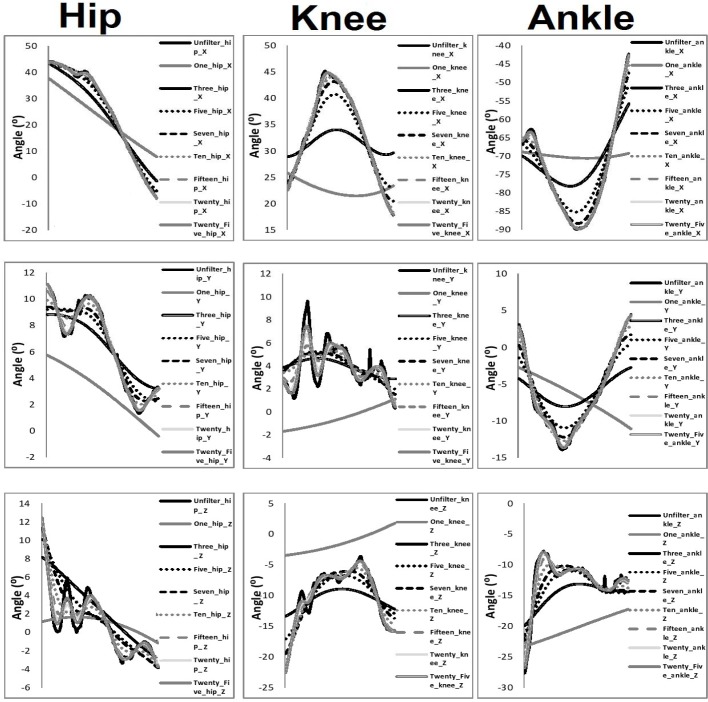
Angular angle displacement waveforms from the hip, knee and ankle as a function of cut-off frequency

**Figure 4:**
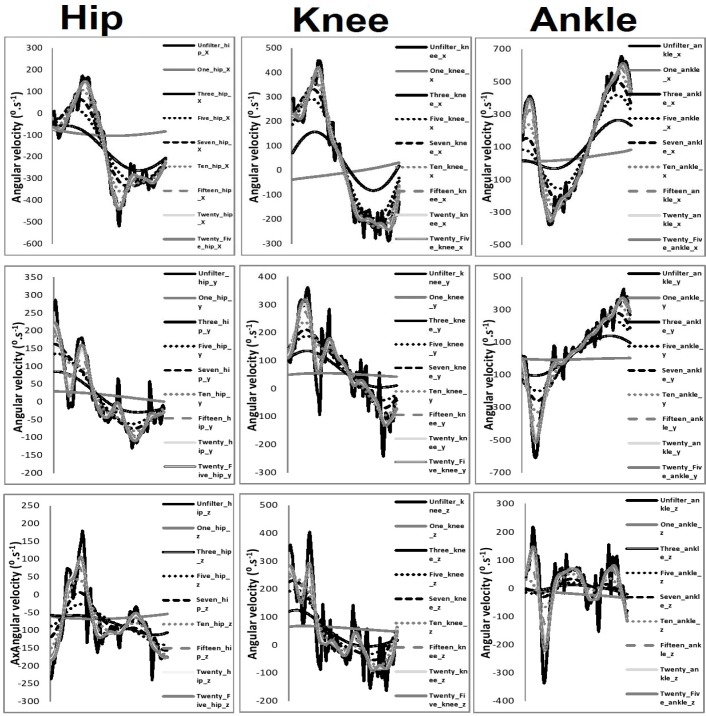
Angular first derivative waveforms from the hip, knee and ankle as a function of cut-off frequency

**Figure 5:**
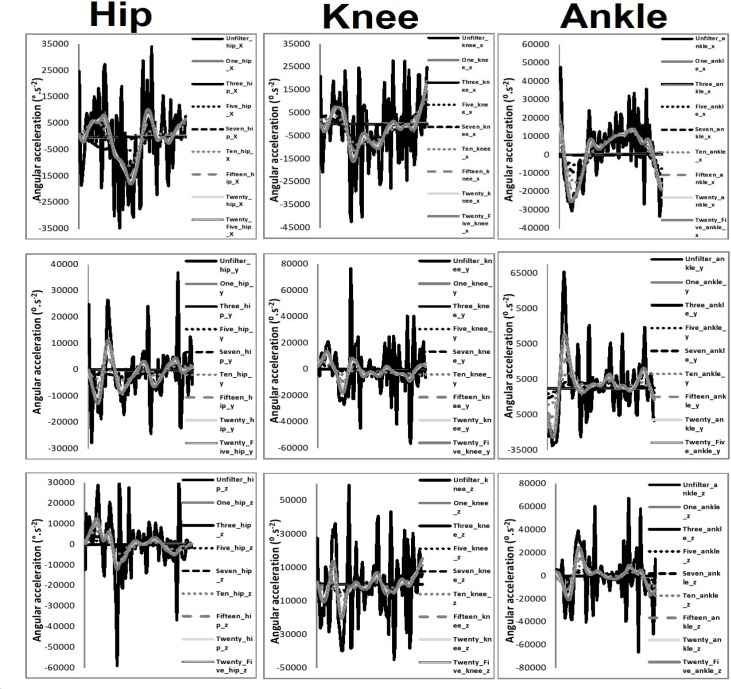
Angular second derivative waveforms from the hip, knee and ankle as a function of cut-off frequency

Tables 1–3 present the intra-class correlation values between filtered and unfiltered waveforms. Figures 3–5 present the mean waveforms and discrete kinematic parameters obtained as a function of cut-off frequency.

### Angular parameters

In the sagittal plane significant main effects were found for the magnitude of peak flexion p≤0.01, η^2^=0.54 and ROM p≤0.01, η^2^=0.89, post-hoc analysis showed that 1 Hz cut-off frequencies differed significantly from the unfiltered, 25, 20 and 15Hz conditions. In the coronal plane a significant main effect was observed for the magnitude of peak adduction p≤0.01, η^2^=0.69, post-hoc analysis showed that 1 and 3 Hz cut-off frequencies differed significantly from the unfiltered, 25, 20 and 15Hz conditions. In the transverse plane a significant main effect was observed for the magnitude of peak external rotation p≤0.01, η^2^ =0.74, post-hoc analysis showed that the 1 Hz cut-off frequency differed significantly from the others.

In the sagittal plane significant main effects were found for the magnitude of peak flexion p≤0.01, η^2^=0.80 and ROM p≤0.01, η^2^=0.53, post-hoc analysis showed that 1 and 3 Hz cut-off frequencies differed significantly from the others. In the transverse plane a significant main effect was observed for the magnitude of peak internal rotation p≤0.01, η^2^ =0.52, post-hoc analysis showed that 1 and 3 Hz cut-off frequencies differed significantly from the others.

In the sagittal plane a significant main effect was observed for the magnitude of ROM p≤0.01, η^2^=0.53 post-hoc analysis showed that the 1 Hz cut-off frequency differed significantly from the others. In the coronal plane a significant main effect was observed for the magnitudes of peak eversion p≤0.01, η^2^=0.45, post-hoc analysis showed that the 1 and 3 Hz cut-off frequencies differed significantly from the others.

### Angular velocity parameters

In the sagittal plane significant main effects were found for the magnitude of peak flexion velocity p≤0.01, η^2^=0.82 and peak extension velocity p≤0.01, η^2^=0.63, post-hoc analysis showed that 1 and 3 Hz cut-off frequencies differed significantly from the others. In the coronal plane significant main effects were found for the magnitude of peak abduction velocity p≤0.01, η^2^=0.62 and peak adduction velocity p≤0.01, η^2^=0.64, post-hoc analysis showed that 1 and 3 Hz cut-off frequencies differed significantly from the others. In the transverse plane significant main effects were found for the magnitude of peak internal rotation velocity p≤0.01, η^2^=0.80 and peak external rotation velocity p≤0.01, η^2^=0.72, post-hoc analysis showed that the 1 Hz cut-off frequency differed significantly from the others.

In the sagittal plane significant main effects were observed at the knee for the magnitude of peak flexion velocity p≤0.01, η^2^=0.56 and peak extension velocity p≤0.01, η^2^=0.53, η^2^=0.72, post-hoc analysis showed that the 1 Hz cut-off frequency differed significantly from the others. In the coronal plane significant main effects were found for the magnitude of peak abduction velocity p≤0.01, η^2^=0.63 and peak adduction velocity p≤0.01, η^2^=0.60 post-hoc analysis showed that 1 and 3 Hz cut-off frequencies differed significantly from the others. In the transverse plane a significant main effect was found for the magnitude of peak internal rotation velocity p≤0.01, η^2^=0.58 post-hoc analysis showed that 1 and 3 Hz cut-off frequencies differed significantly from the others.

In the sagittal plane significant main effects were observed at the ankle for the magnitudes of peak plantarflexion velocity p≤0.01, η^2^=0.61 and peak dorsiflexion velocity p≤0.01, η^2^=0.52, post-hoc analysis showed that 1 and 3 Hz cut-off frequencies differed significantly from the others. Furthermore, significant main effects were found in the coronal plane for the magnitude of peak inversion velocity p≤0.01, η^2^=0.77 and peak eversion velocity p≤0.01, η^2^=0.80, post-hoc analysis showed that 1 and 3 Hz cut-off frequencies differed significantly from the others. In the transverse plane significant main effects were found for the magnitude of peak internal rotation velocity p≤0.01, η^2^=0.62 and peak external rotation velocity p≤0.01, η^2^=0.64, post-hoc analysis showed that 1 and 3 Hz cut-off frequencies differed significantly from the others.

### Angular acceleration parameters

In the sagittal plane a significant main effect was found at the hip for the magnitude of peak acceleration p≤0.01, η^2^=0.99, post-hoc analysis showed that each of the cut-off frequencies differed significantly from one another. In the coronal plane a significant main effect was observed for peak acceleration p≤0.01, η^2^=0.98, post-hoc analysis showed that each of the cut-off frequencies differed significantly from one another. In the transverse plane a significant main effect was observed for the magnitude of peak acceleration p≤0.01, η^2^=0.71, post-hoc analysis showed that each of the cut-off frequencies differed significantly from one another.

In the sagittal plane a significant main effect was observed at the knee for the magnitude of peak acceleration p≤0.01, η^2^=0.92, post-hoc analysis showed that each of the cut-off frequencies differed significantly from one another. In the transverse plane a significant main effect was observed for the magnitude of peak acceleration p≤0.01, η^2^=0.89, post-hoc analysis showed that each of the cut-off frequencies differed significantly from one another.

In the sagittal plane a significant main effect was found at the ankle for the magnitude of peak acceleration p≤0.01, η^2^=0.75, post-hoc analysis showed that each of the cut-off frequencies differed significantly from one another. In the coronal plane a significant main effect was observed for the magnitude of peak acceleration p≤0.01, η^2^=0.96, post-hoc analysis showed that each of the cut-off frequencies differed significantly from one another. In the transverse plane a significant main effect was observed for the magnitude of peak acceleration p≤0.01, η^2^=0.97, post-hoc analysis showed that each of the cut-off frequencies differed significantly from one another.

## Discussion

The aim of the current investigation was to examine the effects of different filter cut-offs and optimal filter techniques on 3-D kinematic waveforms and discrete parameters. This study represents the first to examine the influence of different filters on 3-D displacement and derivate information.

This study examined the influence of nine different cut-off frequencies on the displacement and also first and second derivative joint kinematic parameters. In support of the hypothesis the key observation from the current investigation is that the different cut-off frequencies have a significant influence of discrete kinematic parameters in all three planes of rotation across displacement and derivative waveforms. Qualitative examination of the resultant waveforms obtained as a function of cutoff frequency shows evidence of both under and over smoothing. This therefore emphasizes that notion that selection of an appropriate cut-off frequency is essential in order to achieve empirically meaningful findings and conclusions.

Several techniques currently exist for the determination of an optimal cut-off frequency; the current investigation examined the influence of these techniques on the resultant frequency cutoff. Both the residual and fourier analysis at 95% of the signal power revealed cut-off frequencies of 10 Hz. For joint kinematics from displacement and first derivative measures this cut-off appears to be stable as the waveforms were qualitatively free from noise yet comparative waveform analyses show that the signal is minimally attenuated. Therefore it seems that for the quantification of displacement and first derivative measures the residual and 95% signal power techniques are sufficiently reliable to provide a good estimation for the frequency cut-off. However, when using the spectral technique in which the cut-off frequency is taken at 99% of the signal power the same displacement and first derivative waveforms show clear evidence of noise. Therefore, whilst this cut-off frequency leaves the resultant waveforms minimally attenuated, it appears to be ineffective for the quantification of lower extremity kinematics even at the lower derivatives.

When examining lower extremity kinematics of second derivative angular acceleration curves it is evident that the digital filtering can have a much more pronounced effect on the kinematic waveforms compared to those of the lower derivatives when the same cut-off frequencies are used. This suggests that the signal power of the higher derivative information is contained at a much higher frequency. Like the majority of kinematic analyses the optimal cut-off frequency was calculated by considering only the displacement data which was subjected to be spectral and residual analyses (DAmico and Ferrigno, 1990; Jackson, 1979; Winter et al., 1974; Yu, 1989). The higher derivatives were quantified by filtering the displacement data and then differentiating in the time domain (Damico and Ferrigno, 1990). However as mentioned above, the frequency characteristics of a signal are likely to be different for derivatives of a different order (Yu, 1989). Therefore, it appears that when calculating second derivative kinematics through successive differentiation of displacement data, there is amplification of the noise inherent in apparently smooth displacement curves. This suggests that both residual and spectral analyses of displacement data are not adequate for the determination of cut-off frequencies for higher derivative kinematics.

Time-frequency analysis in which the signal is considered in both time and frequency domains simultaneously has been proposed as an alternative to traditional low pass filtering for derived data. Time frequency analysis techniques typically consist of short-time Fourier transforms and wavelet or Wigner distributions to localize the frequency content of the signals (Stankovic, 2000). However issues do exist with time-frequency based filtering techniques. One of the failings of the short-time fourier transform is its fixed resolution in which the width of the windowing function relates to how the signal is represented. A longer window will give a good frequency resolution but result in a poor temporal resolution, whereas a narrow window function gives good time resolution but poorer frequency resolution. Furthermore, wavelet analyses can also be affected by pseudo-gibbs artefacts which may produce large oscillations in the time signal. Hatze (1981) presented a digital filtering technique in which the optimal filtering factor is quantified separately for each derivative domain. Therefore, instead of first filtering and then differentiating the displacement data, the data are first differentiated and then filtered. This procedure assures a truly optimal regularisation for each of the derivatives (Hatze, 1981). Vaughan (1982) proposed that the displacement data be filtered using a cut-off frequency different from the optimum cut-off frequency derived from displacement data. Both Antonsson and Mann (1985) and Giakas and Baltzopoulos (1997) recommend separate filtering in each derivative domain following estimation of the optimal cutoff using spectral analysis although they did not indicate at what level of the frequency spectrum the cut-off should be implemented. The efficacy of these alternative differentiation and filtering techniques has yet to be evaluated although an understanding of which strategy is more effective will offer a means to improve the performance of existing and future automatic smoothing techniques based on digital filters.

A further interesting observation of the current investigation is that regardless of cut-off frequency kinematic waveforms be more attenuated by the filter in the coronal and transverse planes particularly at the first and second derivatives. This suggests that the smaller more finite rotations outside the sagittal plane may be associated with a different frequency content in predominantly single planar motions such as running. Therefore, it may be that a different cut-off frequency is required for the different planes of rotation although additional work is necessary to confirm this. This observation may be less pronounced in less sagitally dominated movements such as walking or in movements in which the sagittal plane is not the prevailing rotation.

A potential limitation of traditional fourier analyses in kinematic analyses is that the examined signal is truncated to just the stance phase of the gait cycle. Fourier analyses assume that the finite data represents one period of a periodic signal (Walker, 1996). For the FFT, both the time and frequency domain are circular topologies, so that the two endpoints of the time domain signal are interpreted as though they are connected together (Welch, 1970; Harris, 1978; Gautam et al., 1995). Therefore, the finiteness of the sampling record may result in a truncated waveform with different spectral characteristics from the original continuous-time signal. This truncation of the signal can introduce sharp transition changes into the measured data, a phenomenon known as spectral leakage. These sharp transitions are discontinuities and can influence the resultant frequency spectrum. To minimize this effect, we can apply a window function to the measured signal in the time domain. This will make the endpoints of the waveform meet and therefore result in a continuous waveform without sharp transitions. Windows are useful in reducing spectral leakage when using the FFT for spectral analysis, however, because windows are multiplied with the acquired time-domain signal, they introduce distortion effects of their own. The windows therefore change the overall amplitude of the signal.

A further consideration of the current investigation is that a single cut-off frequency was applied to all of the markers. This is commonplace in kinematic analyses but does raise concerns as it is probable that more proximally positioned markers will be associated with a different frequency content than those placed more distally. A select number of researchers have used different frequency cut-offs for markers positioned at different locations (Mills et al., 2011) although this would drastically increase the duration required for data processing it may provide a more meaningful data set. Future work should consider contrasting the kinematic parameters and waveforms when an optimal cutoff has been determined for specific markers against a universal frequency cut-off.

This study examined the influence of a low pass digital filter with different cut-off’s on the same data set, the rationale behind this is that this smoothing mechanism is the most widely utilized in kinematic analyses (Winter et al., 1974; Yu et al., 1999). However, there are many different smoothing methods available in the literature in addition to conventional digital filtering; such as cubic splines (McLaughlin et al., 1977; Soudan and Dierkx, 1979; Zernicke et al., 1976), quantic splines (Wood and Jennings, 1979), polynominal smoothing (Pezzack et al., 1977) and fourier series (Andersson and Bloomfield, 1974). It appears that it would be of practical significance for further analyses to be conducted to examine the effects of different smoothing techniques at different cut-off frequencies on the same data set. This would further enhance knowledge regarding the optimal filter mechanism/ cut-off frequency for the representation of movement analyses.

In conclusion, the results of the current investigation suggest that different cut-off frequencies can have a significant influence on the discrete kinematic parameters. Inspection of the waveform analysis also suggests that for the quantification of 3-D kinematic parameters for displacements and from the lower derivatives that residual and fourier analyses using displacement information from lower extremity markers are able to satisfactorily produce an empirically meaningful cut-off frequency estimate. However future work is necessary in order to determine the most effective filtering technique when examining lower extremity kinematics at the higher derivatives.

## Figures and Tables

**Table 1:** Intraclass correlations for the between filtered and unfiltered displacement waveforms as a function of cut-off frequency

	**25**	**20**	**15**	**10**	**7**	**5**	**3**	**1**
**Hip**								

X (ICC)	1.00	1.00	1.00	1.00	0.999	0.998	0.989	0.904
Y (ICC)	1.00	1.00	1.00	1.00	0.999	0.998	0.977	0.584
Z (ICC)	1.00	0.999	0.997	0.981	0.937	0.881	0.758	0.07

**Knee**								

X (ICC)	1.00	1.00	1.00	1.00	0.998	0.992	0.903	0.130
Y (ICC)	0.998	0.996	0.990	0.978	0.964	0.948	0.814	0.01
Z (ICC)	1.00	1.00	0.999	0.996	0.990	0.978	0.902	0.182

**Ankle**								

X (ICC)	1.00	1.00	1.00	0.999	0.996	0.984	0.885	0.519
Y (ICC)	1.00	1.00	0.999	0.998	0.994	0.986	0.924	0.104
Z (ICC)	1.00	1.00	0.999	0.999	0.996	0.991	0.926	0.213

**Table 2:** Intraclass correlations for the between filtered and unfiltered first derivative waveforms as a function of cut-off frequency

	**25**	**20**	**15**	**10**	**7**	**5**	**3**	**1**
**Hip**								

X (ICC)	0.993	0.992	0.990	0.979	0.956	0.917	0.772	0.043
Y (ICC)	0.984	0.977	0.959	0.923	0.897	0.872	0.774	0.224
Z (ICC)	0.961	0.952	0.937	0.889	0.786	0.619	0.320	0.008

**Knee**								

X (ICC)	0.996	0.994	0.992	0.988	0.982	0.969	0.778	0.340
Y (ICC)	0.972	0.960	0.943	0.911	0.890	0.844	0.685	0.074
Z (ICC)	0.956	0.941	0.917	0.887	0.854	0.809	0.650	0.114

**Ankle**								

X (ICC)	0.997	0.987	0.986	0.983	0.960	0.908	0.684	0.167
Y (ICC)	0.992	0.987	0.981	0.961	0.933	0.894	0.734	0.039
Z (ICC)	0.943	0.909	0.824	0.598	0.273	0.089	0.029	0.007

**Table 3:** Intraclass correlations for the between filtered and unfiltered second derivative waveforms as a function of cut-off frequency

	**25**	**20**	**15**	**10**	**7**	**5**	**3**	**1**
**Hip**								

X (ICC)	0.635	0.603	0.556	0.454	0.225	0.221	0.001	0.001
Y (ICC)	0.644	0.568	0.429	0.209	0.089	0.040	0.008	0.002
Z (ICC)	0.535	0.460	0.381	0.273	0.168	0.078	0.009	0.007

**Knee**								

X (ICC)	0.540	0.503	0.449	0.366	0.304	0.256	0.001	0.001
Y (ICC)	0.457	0.378	0.271	0.147	0.083	0.052	0.001	0.001
Z (ICC)	0.490	0.376	0.243	0.125	0.071	0.039	0.001	0.001

**Ankle**								

X (ICC)	0.874	0.852	0.816	0.742	0.646	0.514	0.009	0.009
Y (ICC)	0.808	0.744	0.620	0.391	0.213	0.110	0.001	0.001
Z (ICC)	0.576	0.516	0.420	0.270	0.154	0.085	0.002	0.001

## References

[b1-jhk-39-25] Anderssen RS, Bloomfield P (1974). Numerical differentiation procedures for non-exact data. Numer Math.

[b2-jhk-39-25] Bartlett R (2007). Introduction to Sports Biomechanics: Analyzing Human Movement Patterns.

[b3-jhk-39-25] DAmico M, Ferrigno G (1990). Technique for the evaluation of derivatives from noisy biomechamcal displacement data using a model-based-bandwidth-selection procedure. Med Biol Eng Comput.

[b4-jhk-39-25] Enoka RM (2008). Neuromechanics of Human Movement.

[b5-jhk-39-25] Harris FJ (1978). On the use of window functions for harmonic analysis with discrete Fourier transform. Proc. IEEE.

[b6-jhk-39-25] Gautam JK, Kumar A RS (1995). Windows: A tool in signal processing. IETE Tech. Rev.

[b7-jhk-39-25] Griffiths IW (2006). Principles of Biomechanics & Motion Analysis.

[b8-jhk-39-25] Hatze H (1981). The use of optimally regularised Fourier series for estimating higher-order derivatives of noisy biomechanical data. J Biomech.

[b9-jhk-39-25] Mills C, Joanna S, Wood L (2011). A protocol for monitoring soft tissue motion under compression garments during drop landings. J Biomech.

[b10-jhk-39-25] Jackson K (1979). Fitting mathematical function to biomechanical data. IEEE Trans Biomed Eng.

[b11-jhk-39-25] Robertson DG, Caldwell G, Hamill J, Kamen G, Whitlesey SN (2004). Research Methods in Biomechanics.

[b12-jhk-39-25] Winter DA (2005). Biomechanics and Motor Control of Human Movement.

[b13-jhk-39-25] McLaughlin TM, Diltman CJ, Lardner TJ (1977). Biomechanical analysis with cubic spline functions. Res Q.

[b14-jhk-39-25] Pezzack JC, Norman RW, Winter DA (1977). Assessment of derivative determining techniques used for motion analysis. J Biomech.

[b15-jhk-39-25] Robertson DGE, Dowling JJ (2003). Design and responses of Butterworth and critically damped digital filters. J Electromyogr Kines.

[b16-jhk-39-25] Sinclair J, Hobbs SJ, Edmundson CJ, Brooks D (2011). Evaluation of kinematic methods of identifying foot strike and toe-off during running. Int J S Sci Eng.

[b17-jhk-39-25] Sinclair J, Taylor PJ, Edmundson CJ, Brooks D, Hobbs SJ (2012). The influence of footwear kinetic, kinematic and electromographical parameters on the energy requirements of steady state running. Mov S Sci.

[b18-jhk-39-25] Sinclair J, Richards J, Taylor PJ, Edmundson CJ, Brooks D, Hobbs SJ (2013a). Three-dimensional kinematics of treadmill and overground running. S Biomech.

[b19-jhk-39-25] Sinclair J, Greenhalgh A, Edmundson CJ, Hobbs SJ (2013b). The efficacy of barefoot and shod running and shoes designed to mimic barefoot running. Footwear Sci.

[b20-jhk-39-25] Smith G (1989). Padding point extrapolation techniques for the Butterworth digital filter. J Biomech.

[b21-jhk-39-25] Soudan K, Dierckx P (1979). Calculation of derivatives and Fourier-coefficients of human motion data, while using spline functions. J Biomech.

[b22-jhk-39-25] Stankovic L (2000). On the time-frequency analysis based filtering. Ann Télécommun.

[b23-jhk-39-25] Vaughan CL (1982). Smoothing and differentiation of displacement - time data: an application of splines and digital filtering. Int J Bio-Med Comp.

[b24-jhk-39-25] Walker JS (1996). Fast Fourier Transforms.

[b25-jhk-39-25] Welch P (1967). The use of fast Fourier transform for the estimation of power spectra: A method based on time averaging over short, modified periodograms. IEEE Trans. on Audio and Electroacoustics.

[b26-jhk-39-25] Winter DA, Sidwall HG, Hobson DA (1974). Measurement and reduction of noise in kinematics of locomotion. J Biomech.

[b27-jhk-39-25] Winter DA (1990). Biomechanics and motor control of human movement.

[b28-jhk-39-25] Wood GA, Jennings LS (1979). On the use of spline functions for data smoothing. J Biomech.

[b29-jhk-39-25] Yu B Determination of the optimum cut-off frequency in the digital filter data smoothing procedure.

[b30-jhk-39-25] Yu B, Gabriel D, Noble L, An KN (1999). Estimate of the optimal cutoff frequency for the butterworth low-pass digital filter. J App Biomech.

[b31-jhk-39-25] Zernicke RF, Caldwell G, Roberts EM (1976). Fitting biomechanical data with cubic spline functions. Res. Q.

